# Evaluation of the Drug–Polymer Compatibility and Dissolution Behaviour of Fenbendazole–Soluplus^®^ Solid Dispersions Prepared by Hot-Melt Extrusion

**DOI:** 10.3390/polym18030333

**Published:** 2026-01-26

**Authors:** Amirhossein Karimi, Gilberto S. N. Bezerra, Clement L. Higginbotham, John G. Lyons

**Affiliations:** 1 The PRISM Research Institute, Technological University of the Shannon: Midlands Midwest, Athlone Campus, University Road, N37 HD68 Athlone, Co. Westmeath, Ireland; a00302530@student.tus.ie (A.K.); clem.higginbotham@tus.ie (C.L.H.); 2 Department of Life and Sciences, Dundalk Institute of Technology, N37 HD68 Dundalk, Co. Louth, Ireland; gilberto.bezerra@dkit.ie

**Keywords:** fenbendazole, solubility enhancement, solid dispersion, Soluplus^®^, hot-melt extrusion

## Abstract

Fenbendazole is an important anti-parasitic medicine widely used in the veterinary field and has recently been considered as a possible anti-cancer agent in humans by some researchers. Fenbendazole encounters challenges in its usage due to its limited aqueous solubility, which consequently impacts its therapeutic efficacy. In this work, an in vitro mechanistic investigation was conducted to evaluate the compatibility, amorphization behaviour and dissolution profile of fenbendazole dispersed in Soluplus^®^ using the solid dispersion approach via hot-melt extrusion. Three different fenbendazole/Soluplus^®^ ratios were formulated and characterised through systematic experimentation. Powder X-Ray Diffraction (PXRD), Differential Scanning Calorimetry (DSC), Scanning Electron Microscopy (SEM), Energy Dispersive X-Ray (EDX) and Fourier Transform Infrared Spectroscopy (FTIR) were employed for thermal, physical, chemical and morphological analyses. The solubility of the drug formulation during a dissolution test was investigated using Ultraviolet–Visible (UV–Vis) spectrophotometric measurements. In vitro dissolution testing in acidic and neutral media was employed as a controlled environment to compare dissolution behaviour among different loadings. The extrudates demonstrated markedly enhanced apparent solubility compared to neat fenbendazole, with the 5% formulation showing the highest dissolution rate (approximately 85% after 48 h). This improvement can be attributed to better wetting properties and drug dispersion within the Soluplus^®^ matrix. This innovative strategy holds promise in surmounting fenbendazole’s solubility limitations, presenting a comprehensive solution to enhance its therapeutic effectiveness.

## 1. Introduction

In the past few years, drug repurpose for existing medications has gained significant attention in pharmaceutical research, such as fenbendazole, which is a commonly used parasiticidal drug in veterinary medicine [1,2,3].

Fenbendazole is categorised as a Biopharmaceutics Classification System (BCS) Class II drug, exhibiting low aqueous solubility but high permeability, leading to low bioavailability and, consequently, restricted overall performance [1,2,4,5,6].

Novel pharmaceutical approaches to increase fenbendazole solubility have been investigated to address its inherent dissolution limitations. Reported approaches include nanosuspensions [7,8,9], solid dispersions [5], co-solvent method [10], lipid-based formulations [11,12] and size reduction [13]. These research approaches are designed to increase fenbendazole’s dissolution behaviour by modifying its solid-state characteristics or enhancing its interaction with aqueous environments.

Fenbendazole has also been reported to exhibit polymorphism, affecting its solid-state and dissolution behaviour. Several studies have described distinct crystal forms or solid-state modifications with measurable differences in apparent solubility [5,10,14]

The literature shows that solid dispersion matrices altered dissolution characteristics of fenbendazole under controlled experimental conditions. These findings are useful for understanding how solid-state properties and drug–polymer interactions may shape dissolution behaviour, although they do not imply a specific therapeutic release requirement [15,16].

The use of solid dispersion formulations incorporating Soluplus^®^ as a key excipient has emerged as a promising strategy to enhance the solubility of poorly water-soluble drugs in pharmaceutical development [17]. Soluplus^®^ is a copolymer which contains amphiphilic segments that allow it to form micellar structures in aqueous settings. This characteristic in solid dispersions is typically associated with enhanced wettability and solubility and reduced crystallinity; however, this work did not propose or examine a specific micelle-mediated release mechanism.

Hot-melt extrusion is extensively utilised for the preparation of amorphous solid dispersions; however, comprehensive studies on fenbendazole remain limited. This study offers a concentrated analysis of fenbendazole–Soluplus^®^ systems by integrating solid-state characterisation with a systematic evaluation of the impact of drug loading on miscibility, amorphization and dissolution behaviour. This study, unlike previous research primarily centred on dissolution enhancement, examines the onset of reduced miscibility within a practically processable loading range, thereby establishing a direct correlation between solid-state characteristics and in vitro dissolution efficacy. This mechanistic viewpoint differentiates the current study from the existing literature on hot-melt-extruded solid dispersions.

This study therefore presents a systematic investigation into the compatibility, solid-state properties and dissolution behaviour of fenbendazole upon its formulation as Soluplus^®^-based solid dispersions. In order to elucidate the impact of polymer–drug interactions, amorphization and dispersion quality on dissolution behaviour, three different drug loadings were prepared as hot-melt-extruded solid dispersions, with the equivalent physical mixtures included only as reference controls under identical in vitro conditions.

## 2. Materials and Methods

### 2.1. Materials

Fenbendazole (molecular weight 299.35 g mol^−1^, with a stated purity of ≥98%) was purchased from Molekula (Darlington, UK), Soluplus^®^ (molecular weight 118,000 g mol^−1^) was purchased from BASF^®^ company with a purity of ≥99% (Ludwigshafen, Germany), hydrochloric acid (HCl) 37%, sodium hydroxide (NaOH, analytical grade, ≥98% purity) pellets and PBS (phosphate-buffered saline) were purchased from Sigma-Aldrich^®^ (Darmstadt, Germany) for pH adjustments in buffer solutions and methanol Chromasolv™ (HPLC grade, ≥99.9% purity) was purchased from Honeywell (Charlotte, NC, USA).

### 2.2. Sample Preparation Method

#### 2.2.1. Physical Mixture (PM)

Physical mixtures of fenbendazole and Soluplus^®^ were prepared at three drug loadings (5%, 10% and 15% *w*/*w*). The selected drug loadings (5–15%) align with the necessary amount for the amorphization of extremely hydrophobic active pharmaceutical ingredients in Soluplus^®^ matrices. Preliminary observations and the existing HME literature indicate that high drug concentrations lead to phase separation or physical instability. The reduced drug concentration was intentionally selected to ensure thorough mixing and to evaluate the efficacy and solubility of fenbendazole within a Soluplus^®^ carrier system. The required quantities of each component were accurately weighed and blended manually using a spatula until homogeneity was attained. The compositions of all formulations are summarised in Table 1.

#### 2.2.2. Continuous Manufacturing Using Hot-Melt Extrusion (HME)

**Figure 1:**
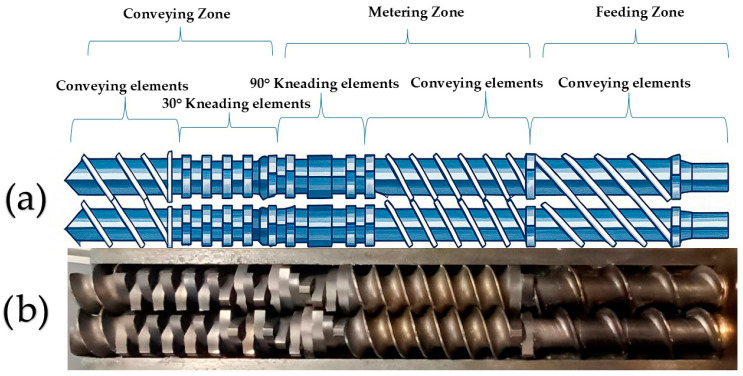
Extruder configuration (**a**) schematic image of screws and (**b**) actual image of the screws.

Before compounding, individual components were weighed and dried in an oven for 24 h at 40 °C to minimise any degradation from absorbed moisture. Then, samples were placed in a sealed Polyethylene bag and manually tumble-blended for 5 min. The blends were processed using a Prism TSE 16 twin-screw co-rotating extruder supplied under the Thermo Fisher Scientific™ /Prism brand, originally part of the Thermo Electron equipment range (Karlsruhe, Germany), and equipped with three heating zones. The extruder was equipped with screws possessing a length/diameter (L/D) ratio of 15:1. The screw configuration consisted of 3 main zones, a feed zone, a conveying zone and a metering zone, as shown in Figure 1.

The compression zone consisted of conveying elements and 10 kneading block elements with a 30° angle. The metering zone also consisted of 10 kneading blocks with a 90° angle and conveying elements.

Physical mixtures were introduced into the system via an automated feeder in a batch size of 100 g and maintained at a feeding rate of 8 g/min. The barrel temperature was held at 110 °C, and the process torque was held constant at 25 ± 5%. The screws’ rotation speed of 110 rpm was applied during the process. The samples collected as filaments. After exiting the die and cooling to room temperature, they were subsequently pelletised using an automated chopper. The formulations are detailed in Table 1, and the abbreviation SOL-E corresponds to Soluplus^®^ extrudate sample.

### 2.3. Characterisation Method

#### 2.3.1. Scanning Electron Microscopy and Energy Dispersive X-Ray Spectroscopy

The samples were broken in liquid nitrogen to expose internal surfaces and mounted on aluminium stubs using conductive carbon tape and sputter-coated with a thin layer of gold for SEM imaging. Uncoated samples were used for EDX analysis. Imaging and semi-quantitative elemental analysis of selected regions were performed using an SEM device produced by TESCAN, MIRA, Inc. (Brno, Czech Republic). This analysis aimed at assessing the morphology of extrudates. At the same time, Energy Dispersive X-Ray spectrometry (EDX) was used to investigate the drug dispersion within the polymeric matrix.

#### 2.3.2. Powder X-Ray Diffraction Spectroscopy (PXRD)

Sample powder was analysed using a PANalytical Aeris Instrument Suite (Malvern Panalytical Ltd., Malvern Panalytical GmbH, Kassel, Germany) with Cu Kα radiation (λ = 1.54078 Å) at 30 kV and 10 mA. Experiments were performed using a Divergence slit 1/8°, a Ni β-filter, 0.04° Soller module and a Pixcel1D-Medipix3 detector. Samples were mounted on a Silicon single-crystal zero-background sample holder. Data were recorded in reflection mode over the range of 5–40° and spinning four rotations per second, using an approximate 0.02° step size and continuous sample spinning at 4 rev·s^−1^.

#### 2.3.3. Differential Scanning Calorimetry (DSC)

Thermal and physical analysis of the samples were performed using the Pyris 6 DSC instrument, a product of PerkinElmer, Inc., Shelton, CT, USA, to analyse the physical properties of our samples. Each sample, weighing approximately 6 mg, was sealed in an aluminium pan for DSC analysis. A nitrogen gas flow rate of 30 ± 1 mL per minute and a heating rate of 10 °C per minute were applied, raising the temperature from 20 °C to 100 °C. After an isothermal process of 1 min at 100 °C, the temperature was reduced to 20 °C with a rate of 10 °C per minute to eliminate any thermal history and moisture. After that, there was another isothermal stage for 1 min at 20 °C. Finally, the samples were reheated from 20 °C to 300 °C. Thermal transitions and the behaviour of the samples were determined using the Pyris Manager Software version 13.3.1.0014. Furthermore, the Origin Pro 2022 software was used to regenerate thermograms and data analysis.

#### 2.3.4. Fourier Transform Infrared Spectroscopy (FTIR)

Sample spectra were acquired utilising a PerkinElmer Spectrum One FTIR spectrophotometer, Shelton, CT, USA, equipped with a universal ATR (Attenuated Total Reflectance) sampling accessory. The scanning range spanned 400–4000 cm^−1^, incorporating four scans for each sample. A constant universal compression force of 85 N was applied during the measurements. Subsequent analysis was performed using Spectrum 10 Module software and Origin Pro 2022.

#### 2.3.5. In Vitro Dissolution Analysis

We used a 2100B paddle apparatus supplied from DISTEK, Inc (North Brunswick, NJ, USA), to perform dissolution experiments. Each extrudate formulation (FEN5-E, FEN10-E, FEN15-E and SOL-E) had approximately 300 ± 10 mg added to 500 mL of dissolution medium, which was maintained at a rate of 37 ± 1 °C and stirred at 50 rpm. This fixed mass of extrudate was selected to enable direct mechanistic comparison of formulations with different drug loadings. The dissolution experiments were not intended to simulate a clinically relevant human dose. The amount of fenbendazole in each sample was either 15, 30 or 45 mg, depending on the actual amount of the drug present. We used neat fenbendazole (15 mg) as a reference. An acidic buffer (pH ≈ 1.2) and a phosphate-based buffer (pH ≈ 6.8) were employed to investigate the effect of pH on dissolution behaviour. At predetermined times (0–2880 min), 3 mL samples were collected and immediately replaced with fresh medium. Extended dissolution times were selected to allow discrimination between formulations and the evaluation of supersaturation stability, rather than to reproduce physiological residence times. Then, the collected samples were filtered using 0.45 μm hydrophobic PTFE filters produced by Fischer Scientific^®^ Waltham, MA, USA. Finally, a Shimadzu UV-1280 spectrophotometer (Shimadzu Corporation, Kyoto, Japan) was used at 288 nm to analyse the samples after they had been filtered. Quantification was performed using a calibration curve generated from standard solutions of fenbendazole. All measurements were conducted in triplicate [15,18]. Although the calibration curve was constructed using standard solutions prepared in methanol to ensure complete solubility of fenbendazole, UV–Vis quantification of dissolution samples was performed directly in the respective aqueous media (pH 1.2 and pH 6.8) at the experimentally determined wavelength of 288 nm. Quantification was based on the linear relationship between absorbance and concentration within the working range, rather than on absolute molar absorptivity values. Blank correction was also applied using Soluplus^®^-containing solutions prepared in the same dissolution media, and the corrected absorbance values were used for all concentration calculations. Non-sink conditions were intentionally selected to enable discrimination between formulations and to evaluate the ability of the polymeric matrix to promote drug solubilisation. The use of surfactants was deliberately avoided, as surfactants can artificially enhance apparent solubility and mask formulation-dependent differences in dissolution behaviour.

## 3. Results

### 3.1. Scanning Electron Microscopy (SEM)

**Figure 2:**
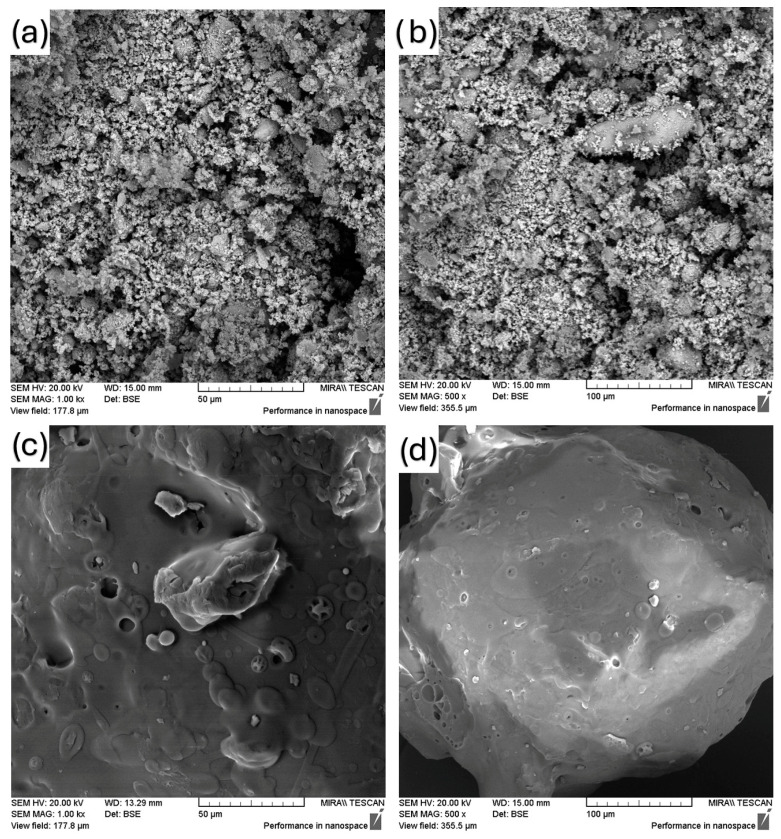
SEM images of (**a**) FEN (1000×), (**b**) FEN (500×), (**c**) SOL (1000×), (**d**) SOL (500×).

**Figure 3:**
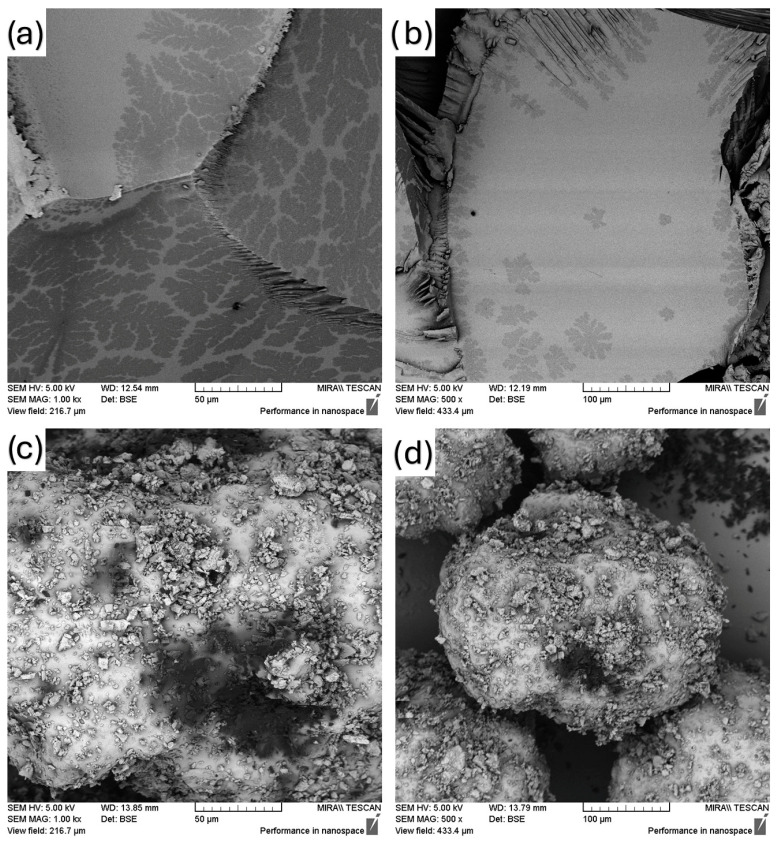
SEM images of (**a**) FEN5-E (1000×), (**b**) FEN5-E (500×), (**c**) FEN5 (1000×), (**d**) FEN5 (500×).

**Figure 4:**
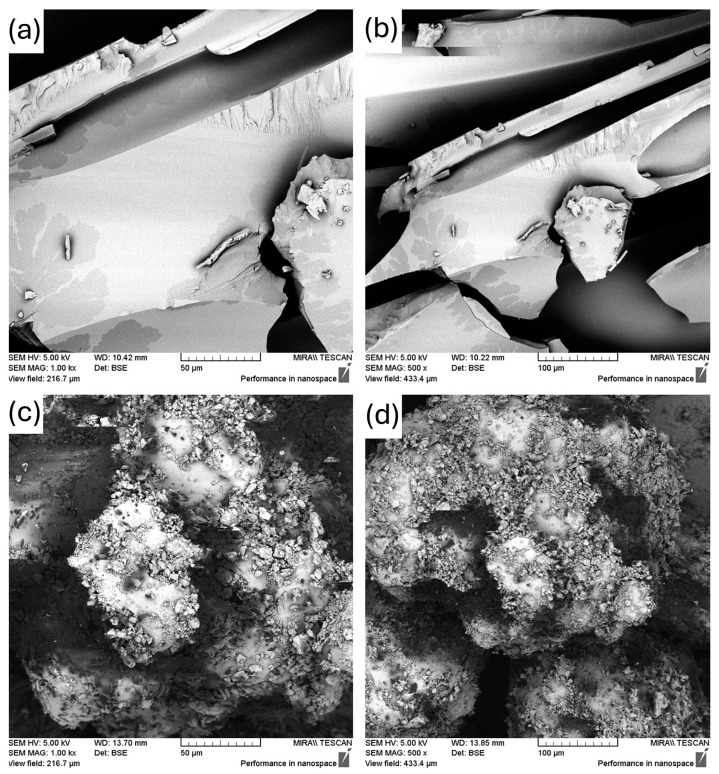
SEM images of (**a**) FEN10-E (1000×), (**b**) FEN10-E (500×), (**c**) FEN10 (1000×), (**d**) FEN10 (500×).

**Figure 5:**
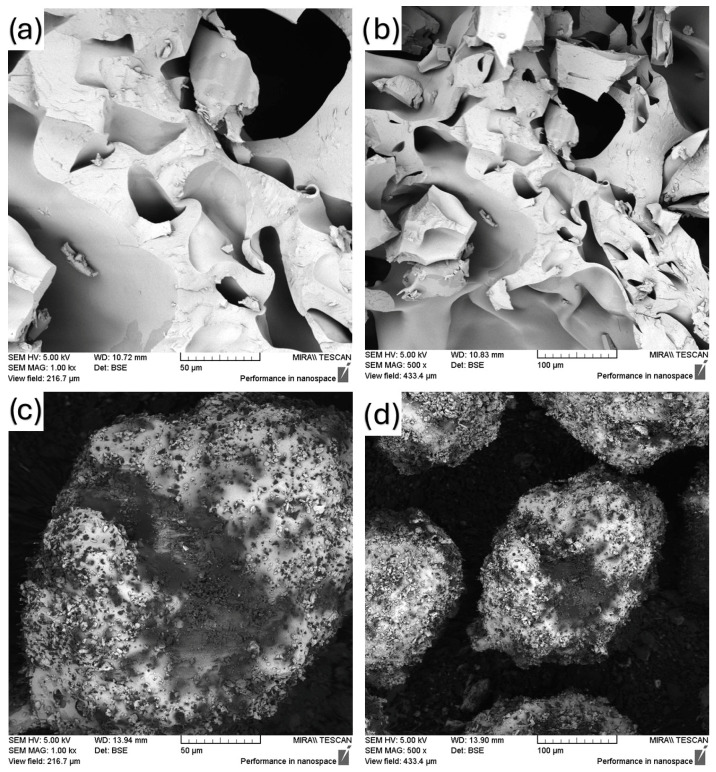
SEM images of (**a**) FEN15-E (1000×), (**b**) FEN15-E (500×), (**c**) FEN15 (1000×), (**d**) FEN15 (500×).

SEM pictures of FEN, SOL, physical mixture samples and the prepared solid dispersion method samples are shown in Figure 2, Figure 3, Figure 4 and Figure 5. Figure 2 shows how the neat materials look in their natural state. Fenbendazole showed a crystalline structure made up of uneven, angular particles with sharp edges, which is what we would expect from its solid-state form. Soluplus^®^, on the other hand, looked like bigger, smoother, amorphous pieces.

The distinct morphological separation of the two components signifies the lack of intrinsic miscibility or interaction prior to processing. These observations create a standard for future comparisons with physical mixtures and hot-melt-extruded samples. Any changes in particle continuity, uniformity or surface texture can be linked to processing effects, not the properties of the raw materials.

SEM images of the physical mixture and extrudate (solid dispersed) samples are shown in Figure 3, Figure 4 and Figure 5, which, respectively, contain 5, 10 and 15% fenbendazole. Figure 3 shows the significant differences between the extrudate with 5% drug loading and the physical mixture.

The physical mixture contains fenbendazole crystals observable as distinct particles adhered to the polymer surface. This indicates that there was no engagement prior to the commencement of the solid dispersion process. The polymer matrix of the extrudate showed a uniform morphology devoid of any discernible crystalline regions. This indicates that the hot-melt extrusion can distribute the drug within the matrix more efficiently. This supports the assertion that employing 5% is the optimal method.

Figure 4 illustrates the distinction between the 10% physical mixtures and its extrudate. After hot-melt extrusion, the fenbendazole crystals were no longer visible on the surface. This means that they had changed into an amorphous form and were now distributed into the polymer matrix. The extrudate, on the other hand, has a rougher surface, small holes and rougher textures compared to the 5% formulation.

Figure 5 illustrates the significant difference between the 15% physical mixture and its corresponding extrudate.

The physical combination exhibits significant drug agglomeration and a distinct two-phase structure, indicating the absence of interaction prior to processing.

The extrudate lacks crystalline domains, indicating that amorphization has occurred. The surface remains rough and has voids. These properties signify limited miscibility and inadequate dispersion at high drug loading, leading to polymer saturation and subsequent microphase separation. The existence of voids and brittle fracture patterns indicates reduced structural integrity. Soluplus^®^ can uniformly incorporate up to 15% fenbendazole. This configuration increases the likelihood of the object being physically unstable, potentially leading to recrystallisation during storage.

In other words, differences in surface morphology would lead to surface area differences, which may affect drug solubility [19,20]. The extrudates were observed with sharp edges and a rougher surface which can be interpreted as the brittleness of the compounds, and, with increasing fenbendazole content, the brittleness increases. At the same time, the greater porosity of the samples that contain more fenbendazole portions can act as macro crazes to make the samples more brittle.

### 3.2. X-Ray Diffraction (XRD)

**Figure 6:**
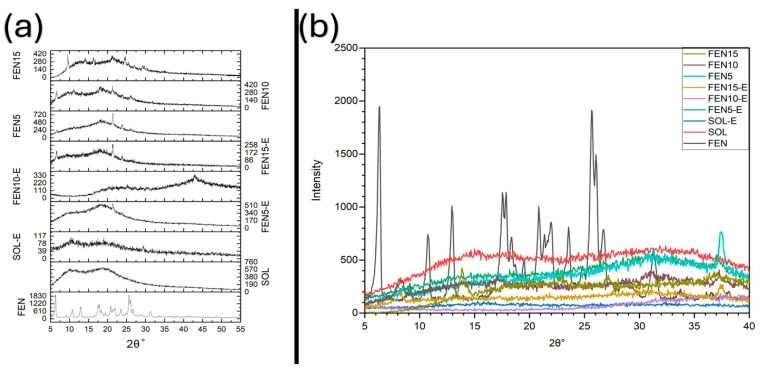
PXRD patterns of fenbendazole (FEN), Soluplus^®^ (SOL), Soluplus^®^ extrudate (SOL-E), physical mixtures (FEN5, FEN10, FEN15) and hot-melt-extruded solid dispersions (FEN5-E, FEN10-E, FEN15-E). (**a**) Stacked diffractograms and (**b**) overlaid patterns showed reduction in crystalline diffraction peaks after extrusion, with weak residual reflections detectable for FEN5-E and FEN15-E. FEN10-E shows a broader and more uniform amorphous halo, indicating improved dispersion at intermediate drug loading.

XRD analysis of FEN displayed clear peaks at characteristic 2θ values (approximately 10°, 14°, 19°, 23°, 26° and 30°), as shown in Figure 6, indicating fenbendazole’s crystalline nature, which is in agreement with known crystallographic properties. In contrast, SOL and SOL-E showed a broad diffuse halo centred around 2θ ≈ 19–22°, typical of amorphous polymers with no long-range order, implying their amorphous structures.

The diffractograms of the physical mixtures (FEN5, FEN10, and FEN15) still showed some peaks, although their intensities decreased obviously. It can demonstrate that simple blending does not alter the crystalline state of fenbendazole prior to processing. In other words, the physical mixtures retain all principal fenbendazole peaks, with intensities according to the quantity of medicine incorporated. Weak residual crystalline reflections remain detectable for FEN5-E and FEN15-E, indicating probable incomplete amorphization at these loadings. FEN10-E shows a broader and more homogeneous amorphous halo, suggesting improved dispersion at intermediate drug loading. However, amorphization does not necessarily imply molecular-level homogeneity; microphase separation may still occur at higher drug loadings. With increased drug loadings, the halo becomes broader and less uniform. The 15% formulation is less prone to mixing and exhibits greater structural variety [15,18]. This non-monotonic evolution of the amorphous halo highlights the existence of an optimal compositional window rather than progressive improvement with increasing drug content.

### 3.3. Differential Scanning Calorimetry (DSC)

**Figure 7:**
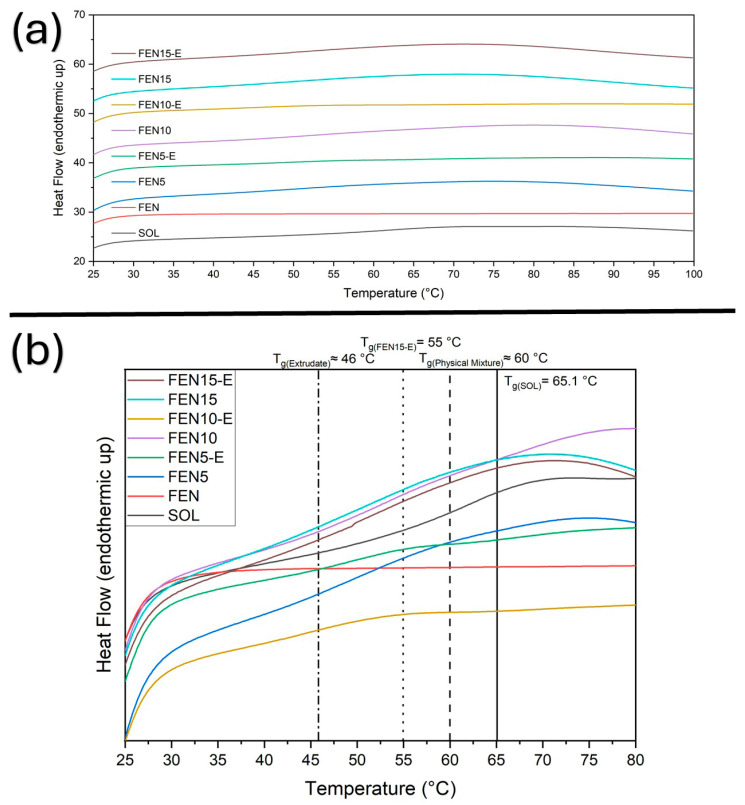
DSC thermograms of fenbendazole (Fen), Soluplus^®^ (Sol) and their physical mixtures and extrudates during the first heating cycle (20–100 °C). (**a**) Full first heating thermograms. (**b**) Expanded view of the T_g_ range illustrating the shift in glass transition temperature for extrudates (Fen5E–Fen15E) compared with Soluplus^®^ (65.1 °C) and physical mixtures.

**Figure 8:**
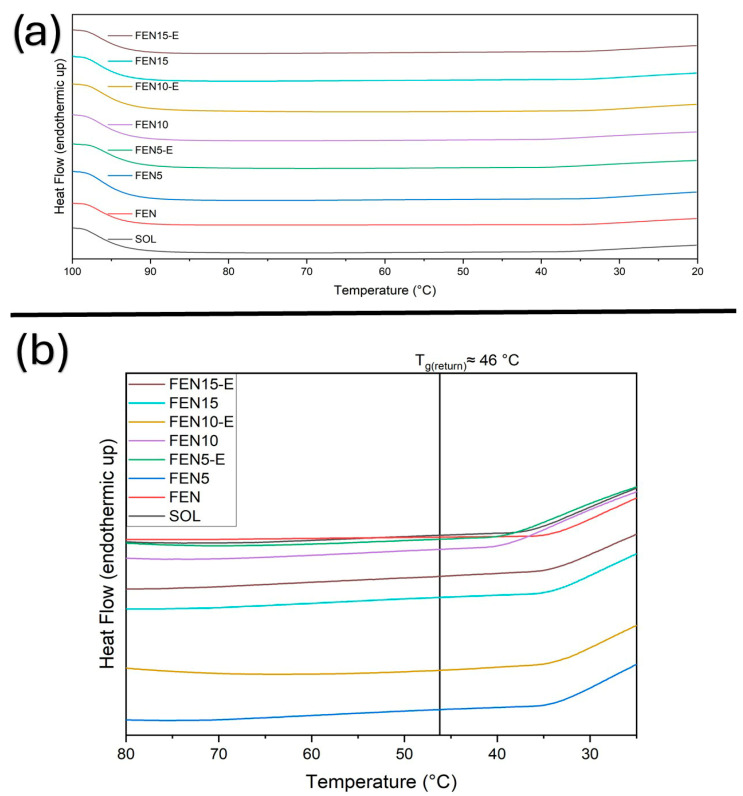
DSC thermograms obtained during the cooling cycle (100–20 °C) for fenbendazole, Soluplus^®^, physical mixtures and extrudates. (**a**) Full thermograms of cooling cycles recorded at a rate of 10 °C·min^−1^. (**b**) Detailed T_g_ range of the samples showed same T_g_ for all the samples except Soluplus (65.1 °C) and fenbendazole (no T_g_).

**Figure 9:**
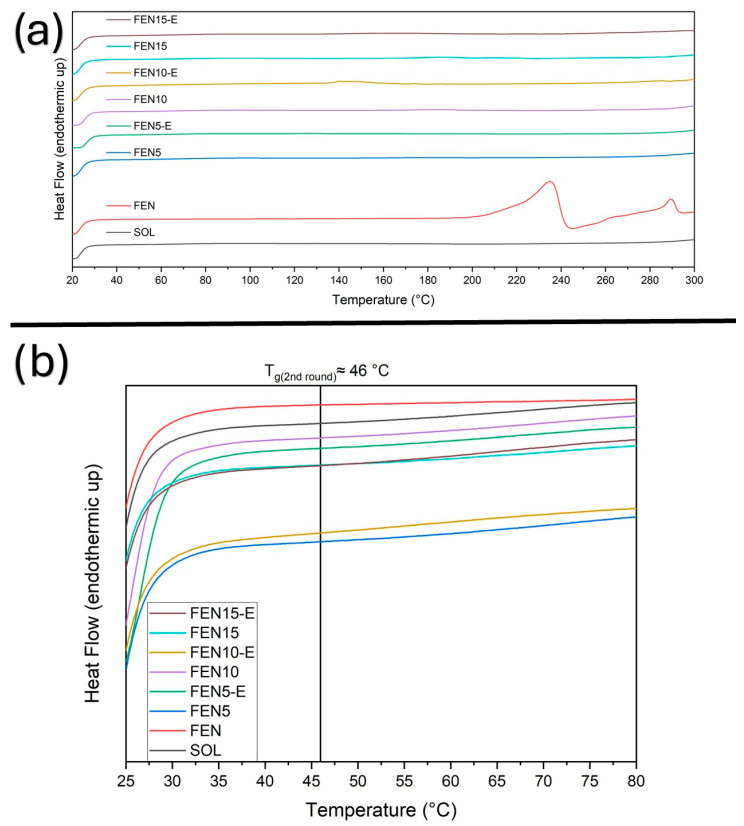
DSC thermograms of fenbendazole, Soluplus^®^, physical mixtures and extrudates during the second heating cycle (20–300 °C). (**a**) Complete thermograms showing the polymer T_g_ and drug-melting range. (**b**) Focused view of the T_g_ range which shows T_g_ remained constant after the first heating cycle for all the physical mixture samples and extrudates. T_g_ of Soluplus remained at 65.1 °C for all three runs of DSC test.

**Figure 10:**
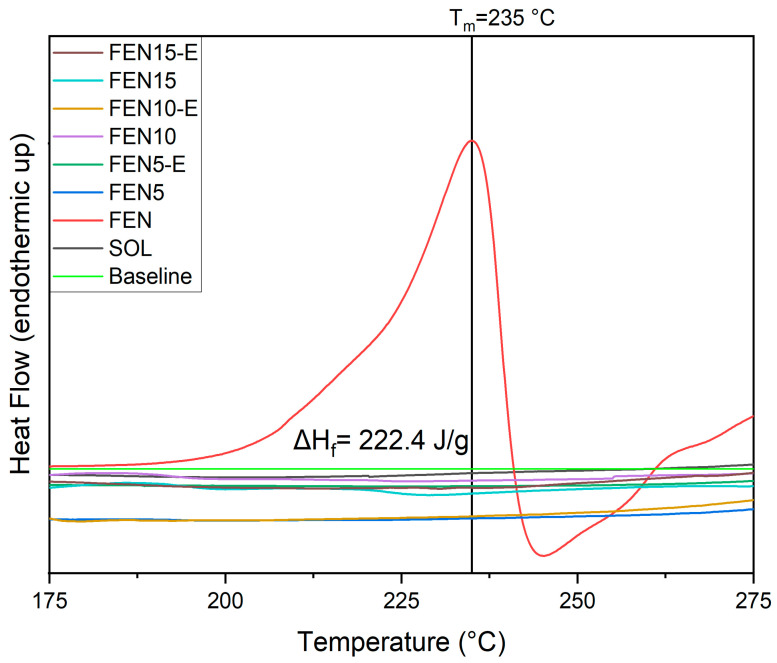
Focused melting temperature range demonstrating the disappearance of the fenbendazole melting endotherm (235 °C, ΔH_f_ = 222.4 J·g^−1^) in the hot-melt extrudates, confirming complete amorphization of the fenbendazole. Disappearance of the same peak in the physical mixture samples confirms dilution of drug within the polymer matrix during the DSC test.

To investigate the thermal and physical behaviour, the DSC technique has been employed. The solid-state characteristics of fenbendazole, Soluplus^®^, their physical mixtures (Fen5, Fen10, Fen15) and the corresponding hot-melt extrudates (Fen5-E, Fen10-E, Fen15-E) have been characterised through DSC. DSC curves were obtained using a heating–cooling–reheating programme. Samples were first heated from 20 to 100 °C to remove thermal history and any absorbed moisture, followed by a 1 min isothermal hold. The temperature was then reduced from 100 to 20 °C, held isothermally for 1 min and subsequently reheated from 20 to 300 °C. Thermograms are shown in Figure 7, Figure 8, Figure 9 and Figure 10, with expanded views of the glass transition temperature (T_g_) and melting temperature (T_m_) regions in different cycles.

During the first heating cycle (Figure 7), Soluplus^®^ exhibited a second-degree transition at 65.1 °C indicating T_g_, which is expected due to its amorphous structure, based on the literature.

On the other hand, fenbendazole did not display any detectable T_g_. All the physical mixtures showed T_g_ around 60 °C, which can be interpreted as a marginal interaction prior to processing. In contrast, Fen5-E, Fen10-E (extrudates) displayed glass transition around 46 °C.

This result indicates the possible effect of the HME on Soluplus^®^ microstructure in the presence of loaded drug. However, Fen15-E, displayed a T_g_ at 55 °C. This figure also shows no moisture in the samples. The reduced T_g_ shift in FEN15-E is consistent with limited drug–polymer miscibility at higher loading, where Soluplus^®^ approaches its saturation capacity. The resulting microphase separation leads to a higher T_g_ compared with the more homogeneous 5% and 10% extrudates. Figure 8 provides additional information about the cooling cycle and the thermal reversibility of all the samples.

The thermograms indicate the same T_g_ at around 46 °C for all the samples, which may show the dilution of fenbendazole in Soluplus^®^ during the first cooling or the isotherm cycle at 100 °C. However, neat fenbendazole does not show any second-degree transition.

In the second heating run (Figure 9), T_g_ showed the same constant number of around 46 °C for the extrudate samples. However, T_g_ of Soluplus^®^ remained constant. It may show that the dilution of fenbendazole in Soluplus^®^ can happen around 100 °C, which is lower than the HME processing temperature in our sample preparation and methodology. According to Figure 9, fenbendazole displayed a sharp melting endotherm peak at 235 °C, corresponding to its crystalline structure.

Even though this peak was also expected in the physical mixtures and even in the extrudates, Figure 10 illustrates no specific peak or even fluctuation in the melting point range. It may confirm the dilution of fenbendazole in Soluplus^®^, in a temperature range much lower than the fenbendazole melting point. Figure 10 also shows the enthalpy of fusion in neat fenbendazole (ΔH_f_ = 222.4 J·g^−1^).

For all hot-melt extrudates, the fenbendazole melting endotherm completely disappeared, indicating that the crystalline drug was converted into an amorphous molecular dispersion during extrusion. The persistence of a single T_g_ and the absence of any endothermic or exothermic events associated with drug recrystallisation upon reheating further confirm the homogeneous amorphous nature of the extrudates.

Overall, the DSC results affirm that HME can effectively produce homogeneous amorphous solid dispersions of fenbendazole in Soluplus^®^. This conversion from a crystalline to an amorphous form is expected to significantly enhance the apparent solubility and dissolution rate of fenbendazole. The thermal transition data derived from the DSC measurements are summarised in Table 2.

### 3.4. Fourier Transform Infrared Spectroscopy (FTIR)

**Figure 11:**
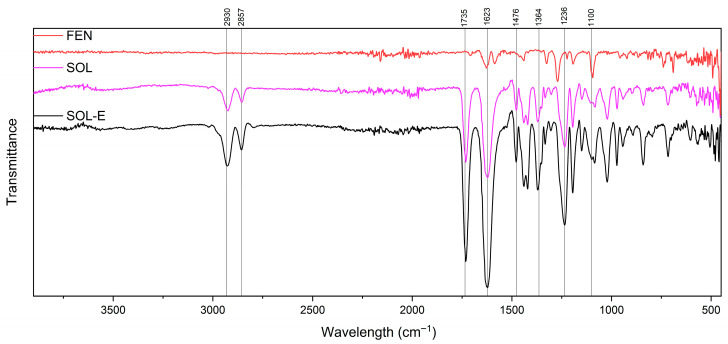
FTIR spectra of fenbendazole (FEN, red), Soluplus^®^ (SOL, pink) and extrudate Soluplus^®^ (SOL-E, black) from 400 to 4000 cm^−1^, showing the characteristic absorption bands of each component and confirming the absence of chemical degradation of Soluplus^®^ after hot-melt extrusion.

Soluplus^®^ is a copolymer consisting of polyvinyl caprolactam, polyvinyl acetate and Polyethylene glycol. Each of these segments has its atoms and bonds with different infrared absorption bands [21,22,23,24]. Interactions and blending of these polymers and the co-polymerisation process may also show more indicative peaks [23], as shown in Figure 11.

In addition, the expected FTIR spectrum of fenbendazole shows peaks corresponding to the benzimidazole ring, carbonyl group, aromatic ring and functional groups.

The presence of polyvinyl caprolactam is distinguished by the caprolactam ring around 1600–1680 cm^−1^, and C–N stretching at 1250–1350 cm^−1^ [21,24,25]. Polyvinyl acetate is recognisable by its acetate groups (CH3COO–) and discernible peaks emerging in the 1730–1740 cm^−1^ range, representative of unsaturated C=O stretching [24,26,27]. Polyethylene glycol, with (C–O–C) bonding, gives rise to observable peaks, typically between 1000 and 1100 cm^−1^, indicative of alcohol C–O stretching vibrations [24,28,29,30].

Benzimidazole ring compounds exhibit distinctive FTIR peaks, such as the carbonyl group (C=O), which can be detected at around 1700–1750 cm^−1^ within the FTIR spectrum [24,31]. Depending on the specific formulation and salt variant of fenbendazole, supplementary functional groups may be determined, potentially yielding unique FTIR peaks [18].

**Figure 12:**
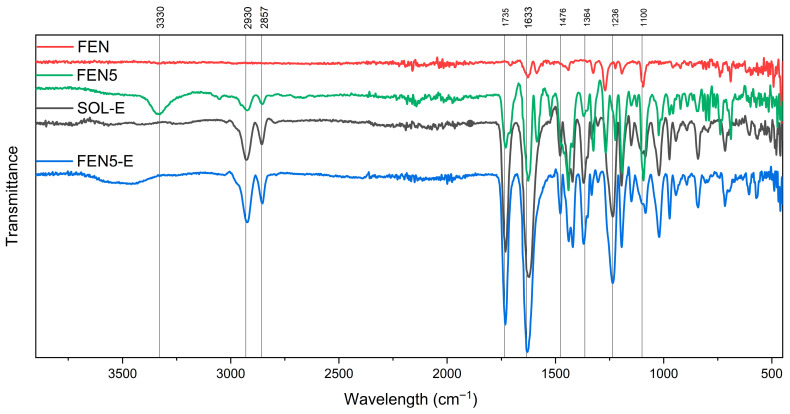
FTIR spectra of fenbendazole (FEN, red), Soluplus^®^ extrudate (SOL-E, black), physical mixture containing 5 percent fenbendazole (FEN5, green) and the same mixture extrudate (FEN5-E, blue) from 400 to 4000 cm^−1^, showing retention of characteristic functional group bands and indicating the absence of chemical modification after extrusion.

The FTIR spectroscopy of FEN, SOL and SOL-E is shown in Figure 11. The spectrum of the extrudate Soluplus^®^ (SOL-E) retained all principal peaks of Soluplus^®^. Figure 12 shows spectra of extrudate Soluplus^®^ (SOL-E), fenbendazole (FEN), their physical mixture and extrudate with a 95:5 mixing ratio. The Fen5-E extrudate exhibited slightly broadened and less intense N–H (≈3300 cm^−1^) and carbonyl (≈1700–1750 cm^−1^) stretching bands compared to FEN5. This broadening suggests hydrogen-bond formation between the amide or imidazole N–H of fenbendazole and the carbonyl or hydroxyl groups of Soluplus^®^.

**Figure 13:**
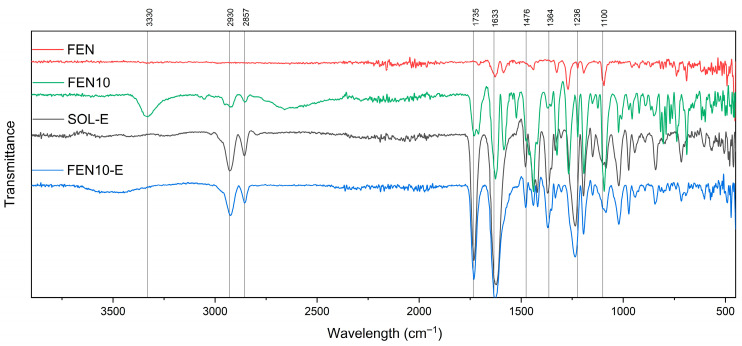
FTIR spectra of fenbendazole (FEN, red), Soluplus^®^ extrudate (SOL-E, black), physical mixture containing 10 percent fenbendazole (FEN10, green) and the same mixture extrudate (FEN10-E, blue) from 400 to 4000 cm^−1^, showing preservation of characteristic absorption bands and indicating no chemical modification following extrusion.

The FEN10-E spectrum showed the same trend as FEN-5E with reduced intensity of aromatic ring vibrations around 1470 cm^−1^ (Figure 13).

**Figure 14:**
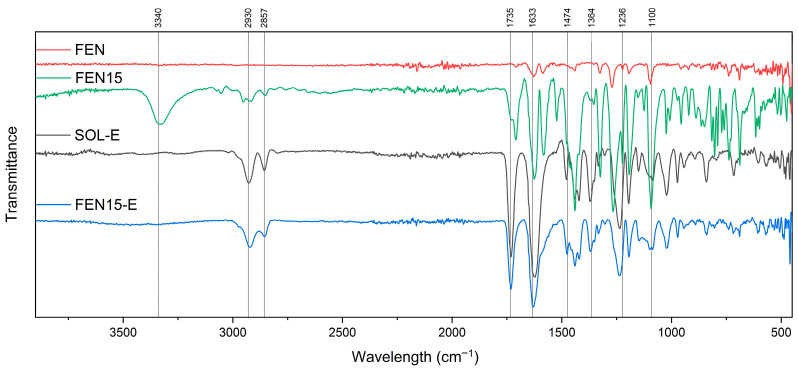
FTIR spectra of fenbendazole (FEN, red), Soluplus^®^ extrudate (SOL-E, black), physical mixture containing 15 percent fenbendazole (FEN15, green) and the same mixture extrudate (FEN15-E, blue) from 400 to 4000 cm^−1^, illustrating no chemical reaction after HME.

The absence of new bands and the observed attenuation of fenbendazole peaks indicate strong molecular interactions rather than chemical modification. Figure 14 shows spectra related to FEN15-E, showing a broad –H stretching band centred around 3340 cm^−1^, while the same peak in the spectra related to the physical mixture is sharper than the extrudate. At the same time, benzimidazole ring peaks (related to C=C) near 1470 cm^−1^ remained noticeable. These changes may confirm enhanced hydrogen bonding and improved miscibility. The spectra of all extrudates showed no new peaks, which indicates that fenbendazole remained chemically stable and unchanged after the extrusion process.

In summary, Figure 12, Figure 13 and Figure 14 show the FTIR spectrum of different formulations in the physical mixture and extrudate state. The compatibility may indicate that there is no chemical reaction between the Soluplus^®^ chains and the fenbendazole chemical structure [15,18,32]. A summary of more driven data from FTIR analysis is presented in Table 3. This table shows the important indicators and differences in chemical structures.

### 3.5. Energy Dispersive X-Ray Spectroscopy

EDX was employed to confirm the presence and relative distribution of fenbendazole within the analysis area, using sulphur as an elemental marker, since Soluplus^®^ does not contain sulphur in its chemical structure. This analysis was intended to provide semi-quantitative insight into drug dispersion following hot-melt extrusion, rather than to confirm molecular-level miscibility.

**Figure 15:**
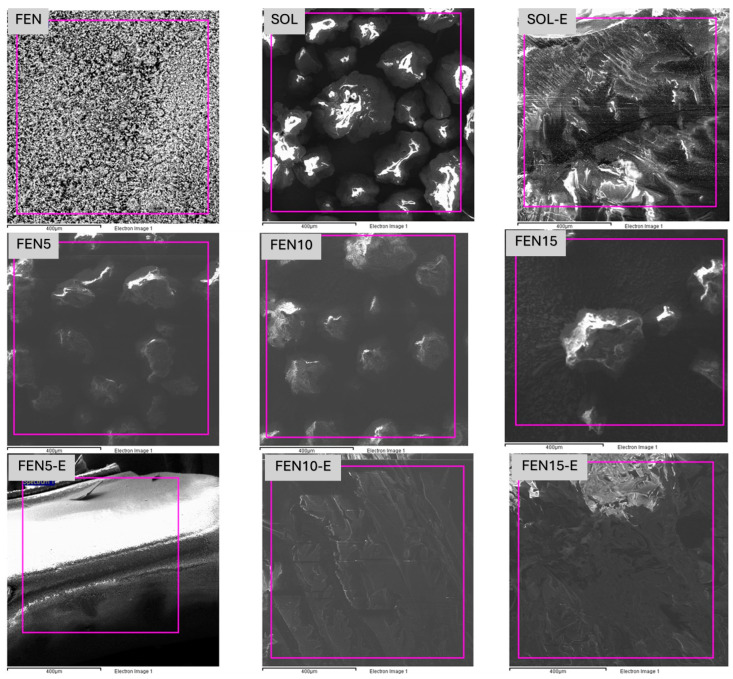
SEM images with corresponding EDX spectra of selected regions, to investigate the elemental distribution in physical mixtures and extruded formulations.

Our analysis revealed that sulphur was present in all the samples except SOL and SOL-E. The analysis area for each formulation is shown in Figure 15. EDX measurements were performed on specific areas of the sample surface rather than as full elemental mapping.

The EDX spectra confirmed the presence of sulphur in all fenbendazole-containing samples, while no sulphur signal was detected in neat Soluplus^®^ or its extrudate (SOL-E), supporting the chemical integrity of the polymer during processing. Differences in sulphur content between physical mixtures and extruded samples were observed (Table 4), reflecting changes in the local elemental composition after hot-melt extrusion.

The differences between the expected sulphur portion and the analysed sulphur portion in the physical mixture samples may be indicative of a lack of adequate dispersion. However, in terms of solid dispersed samples, the difference can be related to the suboptimal dispersion, which was expected according to the literature [15,18]. Additionally, this discrepancy is attributed to the surface-sensitive and semi-quantitative nature of EDX analysis. Such deviations are expected when EDX is applied to heterogeneous polymer–drug systems and do not indicate degradation or loss of fenbendazole during processing.

### 3.6. In Vitro Dissolution Behaviour

**Figure 16:**
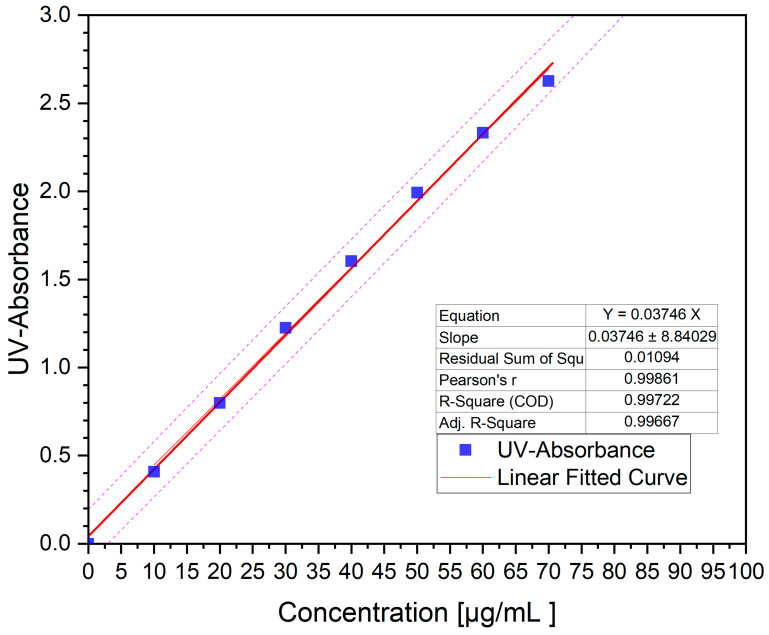
Calibration curve of fenbendazole dissolved in methanol. The pink dashed lines represent the confidence band around the linear regression fit.

To create the initial stock standard solution and prepare the calibration curve, 20 mg of fenbendazole was dissolved in 200 mL of methanol, resulting in a 100 μg/mL standard solution concentration. This stock solution was diluted to generate a range of final concentrations, specifically 10, 20, 30, 40, 50, 60 and 70 μg/mL. By plotting a calibration curve (Figure 16) correlating absorbance to concentration, an impressive R^2^ value of 0.99722 was achieved.

Although the calibration standards were prepared in methanol, the linearity of absorbance at 288 nm was used for the quantification of samples measured in aqueous media, where the same wavelength was selected based on preliminary UV scans. The dissolution behaviour of neat fenbendazole, physical mixtures and hot-melt-extruded samples was assessed in two media: an acidic buffer (pH 1.2) and a phosphate-based buffer (pH 6.8). The experiments were conducted in triplicate, and the data obtained were analysed to elucidate the formulation’s drug dissolution kinetics and overall performance. All UV–Vis data were blank-corrected using Soluplus^®^ reference solutions to eliminate polymer-related background absorption.

The drug dissolution was evaluated over 48 h (2880 min) in both media. These dissolution analysis times were selected solely to enable mechanistic comparison between formulations under constant conditions and were not intended to represent physiological gastric residence. The study does not aim to model in vivo gastrointestinal transit. The results revealed that the ultimate dissolution profile will increase for solid dispersed formulations in the acidic medium. FEN5-E dissolves 85.02% of the loaded drug average, while this number is 67.08% for FEN10-E and for FEN15-E it is 53.27%. A significant improvement is observable compared to the FEN ultimate dissolution amount of 19.98%. Since physical mixtures showed no evidence of drug–polymer interaction or amorphization in FTIR or SEM analyses, dissolution testing was limited to the pure drug and the extruded formulations.

**Figure 17:**
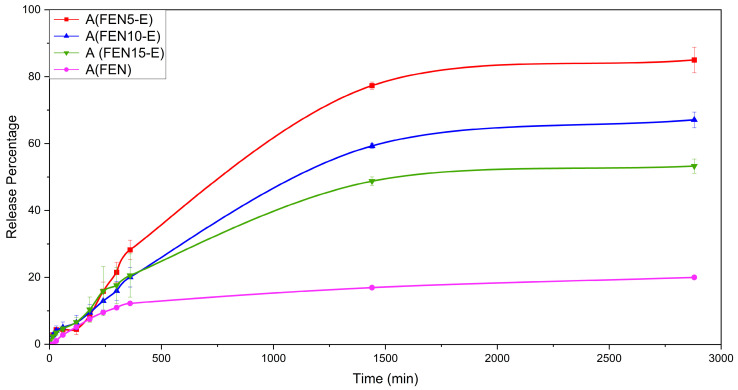
The dissolution profile of FEN and extrudate samples in an acidic environment in 48 h (2880 min).

Consequently, results showed increased solubility in all the extrudate samples, specifically FEN5-E. Additionally, dissolution behaviour improvement decreased with increasing the fenbendazole portion in the formulation. The initial dissolution at the first hour even showed a slight increase. However, as time progressed, this increase became more pronounced and significant. Figure 17 illustrates the drug dissolution profile over time, while “A” stands for acidic environment.

**Figure 18:**
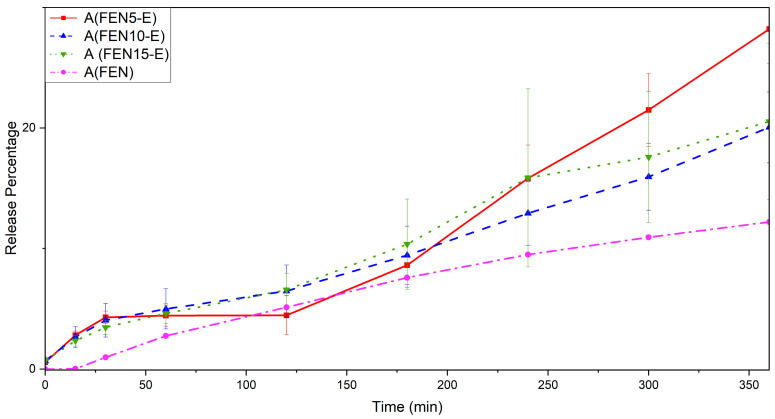
The dissolution profile of FEN and extrudate samples in an acidic environment in the first 6 h.

Figure 18 shows the dissolution behaviour in the first 6 h, while “A” stands for acidic buffer solution. It shows that, in the first 3 h, all the solid dispersed samples have the same dissolution rate. It can probably be because of non-homogenous samples produced by solid dispersion. Accordingly, the 6 h and 48 h acidic dissolution profiles should be interpreted as comparative in vitro data rather than as biorelevant simulations of gastric exposure.

**Figure 19:**
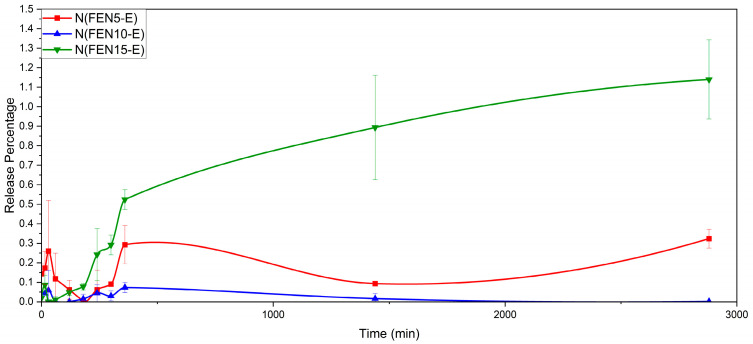
The dissolution profile of FEN and extrudate samples in a neutral environment in 48 h (2880 min).

On the other hand, the results showed that fenbendazole does not dissolve in the neutral medium (pH = 6.8). In addition, in solid dispersed samples with increasing fenbendazole content, the solubility shows a slight increase. However, this increase is not considerable and does not follow any specific trend. Figure 19 shows the dissolution behaviour of extrudate formulations, while “N” stands for neutral environment.

## 4. Discussion

### 4.1. Effective Factors on Drug Dissolution Rate

The impact of pH and concentration were investigated by other groups to understand better the factors influencing the drug dissolution [15,18,33]. The findings indicate that, with decreasing pH, solubility increases, and changing pH may alter the dissolution mechanism. According to the previous results, the concentration of fenbendazole can be effective through different trends in dissolution behaviour. At lower pH values (acidic media), it affects it oppositely, while in the neutral environment, they have a direct but not considerable relation.

### 4.2. Dissolution Mechanism

To design drug delivery systems, it is crucial to understand how drugs are dissolved from formulations. The dissolution behaviour of fenbendazole from the Soluplus^®^ matrix was evaluated using five kinetic models (zero-order, first-order, Higuchi, Korsmeyer–Peppas and Peppas–Sahlin) to identify the predominant dissolution mechanism. Some criteria, such as R^2^ adjusted (the highest), AIC (the lowest) and MSC (the highest), are used [34] to gain insights into the factors that govern the dissolution of fenbendazole.

A first-order release model provided the most accurate description of the dissolution kinetics for the FEN5-E formulation. This result implies that the initial loading of fenbendazole in the formulation is critical in determining the amount of medication dissolved. According to a first-order kinetic mechanism, the starting concentration directly affects how quickly fenbendazole is dissolved into the dissolving solvent.

The Peppas–Sahlin model also provided a good correlation (R^2^ > 0.95), with the k_1_ values approaching zero and acceptable k_2_ values for all the FEN5-E, FEN10-E and FEN15-E samples, suggesting that polymer relaxation and erosion played dominant roles over Fickian diffusion. Such non-Fickian behaviour implies a combined mechanism involving both diffusion and matrix relaxation [35].

The dissolution rate of fenbendazole is greatly aided by the physicochemical structure of the polymer–drug matrix within the solid dispersion formulation. In solid dispersed samples processed by hot-melt extrusion, the drug distribution and dispersion quality, the amorphization and the polymer’s molecular mobility may affect diffusion, relaxation or erosion, which dominates the dissolution profile [35]. The better correlation of the FEN5-E dissolution with the first-order model may suggest that drug diffusion is concentration-dependent, which is typical for matrices with partial phase separation or heterogeneous drug dispersion [35,36]. This behaviour is consistent with the morphological observations by SEM, where a relatively homogeneous but partially phase-separated microstructure was evident. Such morphology allows progressive diffusion of fenbendazole through the Soluplus^®^ network as the polymer swells and relaxes. In contrast, the Peppas–Sahlin model shows that polymer chain relaxation and erosion play a dominant role in the dissolution mechanism, compared to concentration. This fact aligns with the viscoelastic and amphiphilic characteristics of Soluplus^®^ [35,36,37,38]. This interpretation may be supported by the DSC and XRD results, which confirmed the complete amorphization of fenbendazole within the polymeric matrix, and by the FTIR findings, showing strong hydrogen-bond interactions between the fenbendazole and Soluplus^®^. Consistent with the scope defined in the Introduction, the dissolution behaviour observed in this study is interpreted primarily in terms of amorphization, enhanced wettability and drug–polymer miscibility. All the information and calculated parameters are shown in Table 5.

### 4.3. Comparison with Previous Studies

Compared to these specific in vitro drug dissolution measurements, the relevant literature showed different dissolution behaviours. For example, Bezerra et al. showed that, in the same method with Poly Caprolactone (PCL) and Polyethylene Oxide (PEO), there is an initial burst dissolution for fenbendazole in the first 8 h [15]. The primary dissolution mechanism for PEO is categorised as anomalous, meaning it relies on processes like swelling, drug diffusion and matrix erosion. Higher concentrations of PEO result in faster drug dissolution rates. Conversely, PCL, being a hydrophobic polymer, is responsible for slowing down the drug dissolution rates, which is close to another study by Quinten et al. [15,18,39]. In another study, Jin et al. used Soluplus^®^ to increase the solubility and enhance the dissolution of fenbendazole through the solution method and to make micelles [40]. This research showed a sustained release through 14 days but in the same ultimate percentage compared to the current study. This comparison contributes to a broader understanding of our findings within the context of existing research. In another study, Melian et al. showed a fast release of fenbendazole in formulations based on Poloxamer 188 (P188) and Poloxamer 407 (P407) through solid dispersion, following the Lumped model [41]. Analysis of this specific mathematical model revealed that the processing method was the key factor affecting fenbendazole release. The methodology effect is more significant than the effect of the type or amount of polymer used in the formulation.

## 5. Conclusions

By implementing solid dispersion technology in conjunction with Soluplus^®^, the solubility of fenbendazole in acidic environments can be significantly improved. Converting fenbendazole into an amorphous state within the solid dispersion matrix could be the main reason for this phenomenon within the investigated drug loading range. The presence of Soluplus^®^ further enhances solubility improvement through efficient drug–polymer interactions and improved wettability.

Comprehensive physical, chemical and microscopic analysis confirmed the success of this innovative strategy. Notably, in vitro dissolution evaluation showed a pronounced increase in drug dissolution compared to the neat drug, with cumulative dissolution rising from approximately 20% for fenbendazole to approximately 85% for the 5% Soluplus^®^-based solid dispersion under acidic conditions. This improvement is due to the converted crystalline structure of fenbendazole to an amorphous state within the Soluplus^®^ matrix without implying the determination of an absolute polymer saturation threshold.

## Figures and Tables

**Table 1:** Samples formulations compositions and preparation method.

	Samples	FEN5	FEN10	FEN15	FEN5-E	FEN10-E	FEN15-E	SOL-E	FEN	SOL
Characteristics										
Fenbendazole (gram)		5	10	15	5	10	15	0	5	0
Soluplus^®^ (gram)		95	90	85	95	90	85	100	0	5
Preparation Method *		PM	PM	PM	HME	HME	HME	HME	Neat	Neat

* In the preparation method, “PM” refers to the physical mixture, “HME” denotes the hot-melt extrusion process and “Neat” represents the pure, unprocessed material.

**Table 2:** Thermal characteristics of the neat samples, physical mixtures and extrudates obtained from DSC analysis.

Sample	T_g_ (1st Heating Cycle) °C	T_g_ (1st Cooling Cycle) °C	T_g_ (2nd Heating Cycle) °C	T_m_ (2nd Heating Cycle) °C
FEN	-	-	-	235 (in a range)
SOL	65.1	65.2	65.1	-
FEN5	60.7	46.9	46.9	-
FEN5-E	46.1	46.0	45.8	-
FEN10	61.2	46.5	46.6	-
FEN10-E	46.8	45.8	46.0	-
FEN15	59.8	55.7	55.3	-
FEN15-E	55.0	54.6	52.3	-

**Table 3:** Presence of different chemical bonds and functional groups in the neat samples, physical mixtures and extrudates.

	Bonds, Wavelength Range cm^−1^	C–S Thioether 700–750	C–O–C (Ether) 1000–1100	N–H Bending (Amide) 1540–1560	C=C Aromatic Ring Stretching 1450–1600	C=O Stretching (Carbomate) 1700–1720	C=O (Ester) 1730–1740	N–H –H Hydrogen Bond 3200–3600
Sample								
FEN		☑	–	☑	☑	☑	–	☑ (Negligible)
SOL		–	☑	☑	–	–	☑	–
FEN5		☑	☑	☑	☑	☑	☑	☑ (Obvious)
FEN5-E		☑	☑	☑	☑	☑	☑	☑ (Broad)
FEN10		☑	☑	☑	☑	☑	☑	☑ (Obvious)
FEN10-E		☑	☑	☑	☑	☑	☑	☑ (Broad)
FEN15		☑	☑	☑	☑	☑	☑	☑ (Obvious)
FEN15-E		☑	☑	☑	☑	☑	☑	☑ (Broad)

“☑” shows the presence of the chemical bonds while “–“shows their absence.

**Table 4:** EDX semi-quantitative elemental analysis *.

	Elements%	Carbon	Oxygen	Sulphur	Expected Sulphur	Others	Total
Samples							
FEN		59.37	12.90	9.29	9.29	18.44	100.00
SOL		67.61	32.39	-	-	-	100.00
SOL-E		70.80	29.12	-	-	0.08	100.00
FEN5-E		64.34	35.29	0.09	0.5	0.27	100.00
FEN10-E		69.50	29.95	0.55	1	-	100.00
FEN15-E		64.26	35.58	0.16	1.5	-	100.00
FEN5		67.65	31.72	0.63	0.5	-	100.00
FEN10		68.71	29.72	1.57	1	-	100.00
FEN15		71.71	24.84	3.45	1.5	-	100.00

* The elemental compositions reported in Table 4 represent semi-quantitative values obtained from selected surface regions and should not be interpreted as bulk composition measurements.

**Table 5:** Dissolution models and parameters *.

Release Model	Equation	Parameter	FEN5-E	FEN10-E	FEN15-E
Zero-order	$F=k_{0}\cdot t$	R^2^ _adjusted_	0.83	0.84	0.71
		AIC	81.88	75.44	76.09
		MSC	1.66	1.70	1.19
First-order	$F=100\cdot (1-e^{-k_{1}\cdot t})$	R^2^ _adjusted_	0.97	0.95	0.85
		AIC	59.63	59.58	67.75
		MSC	3.68	3.15	1.95
Higuchi	$F=k_{H}\cdot t^{0.5}$	R^2^ _adjusted_	0.90	0.92	0.93
		AIC	76.06	67.11	61.42
		MSC	2.19	2.46	2.53
Korsmeyer-Peppas	$F=k_{KP}\cdot t^{n}$	R^2^ _adjusted_	0.93	0.95	0.94
		AIC	72.95	63.21	60.14
		MSC	2.47	2.82	2.65
		N	0.64	0.63	0.55
Peppas-Sahlin	$F=k_{1}\cdot t^{m}+k_{2}\cdot t^{2m}$	R^2^ _adjusted_	0.95	0.96	0.97
		AIC	65.61	55.30	45.91
		MSC	3.14	3.53	3.94
		M	0.47	0.45	0.63
		k_1_	−6.47	−3.57	−1.84
		k_2_	3.09	1.73	1.08

* F represents the fraction of drug released in time t, k_0_ is the zero-order release constant, k_1_ is the first-order release constant, k_H_ is the Higuchi release constant, k_KP_ is the release constant incorporating structural and geometric characteristic of the dosage form, n is the diffusional exponent indicating the mechanism of drug release, k_1_ is the constant related to the Fickian kinetics, k_2_ is the constant associated with Case-II relaxation kinetics and the diffusional exponent, denoted as m, is a parameter for a device of any shape that regulates the release rate [34].

## Data Availability

The original contributions presented in this study are included in the article. Further inquiries can be directed to the corresponding author.

## References

[1] Dogra N., Kumar A., Mukhopadhyay T. (2018). Fenbendazole Acts as a Moderate Microtubule Destabilizing Agent and Causes Cancer Cell Death by Modulating Multiple Cellular Pathways. Sci. Rep..

[2] Park D., Lee J.-H., Yoon S.-P. (2022). Anti-Cancer Effects of Fenbendazole on 5-Fluorouracil-Resistant Colorectal Cancer Cells. Korean J. Physiol. Pharmacol..

[3] Pushpakom S., Iorio F., Eyers P.A., Escott K.J., Hopper S., Wells A., Doig A., Guilliams T., Latimer J., McNamee C. (2019). Drug Repurposing: Progress, Challenges and Recommendations. Nat. Rev. Drug Discov..

[4] Esfahani M.K.M., Alavi S.E., Cabot P.J., Islam N., Izake E.L. (2022). β-Lactoglobulin-Modified Mesoporous Silica Nanoparticles: A Promising Carrier for the Targeted Delivery of Fenbendazole into Prostate Cancer Cells. Pharmaceutics.

[5] Melian M.E., Paredes A., Munguía B., Colobbio M., Ramos J.C., Teixeira R., Manta E., Palma S., Faccio R., Domínguez L. (2020). Nanocrystals of Novel Valerolactam-Fenbendazole Hybrid with Improved in Vitro Dissolution Performance. AAPS PharmSciTech.

[6] Esfahani M.K.M., Alavi S.E., Cabot P.J., Islam N., Izake E.L. (2021). PEGylated Mesoporous Silica Nanoparticles (MCM-41): A Promising Carrier for the Targeted Delivery of Fenbendazole into Prostrate Cancer Cells. Pharmaceutics.

[7] Agrawal Y., Patel V. (2011). Nanosuspension: An Approach to Enhance Solubility of Drugs. J. Adv. Pharm. Technol. Res..

[8] Li X., Gu L., Xu Y., Wang Y. (2009). Preparation of Fenofibrate Nanosuspension and Study of Its Pharmacokinetic Behavior in Rats. Drug Dev. Ind. Pharm..

[9] Ravichandran R. (2010). Preparation and Characterization of Albendazole Nanosuspensions for Oral Delivery. Int. J. Green Nanotechnol. Biomed..

[10] Yang Z., Shao D., Zhou G. (2020). Analysis of Solubility Parameters of Fenbendazole in Pure and Mixed Solvents and Evaluation of Thermodynamic Model. J. Chem. Thermodyn..

[11] Mohsin K. (2012). Design of Lipid-Based Formulations for Oral Administration of Poorly Water-Soluble Drug Fenofibrate: Effects of Digestion. AAPS PharmSciTech.

[12] Niederquell A., Dujovny G., Probst S.E., Kuentz M. (2019). A Relative Permittivity Approach for Fast Drug Solubility Screening of Solvents and Excipients in Lipid-Based Delivery. J. Pharm. Sci..

[13] Karimi A., Barea P., Benito-Román Ó., Blanco B., Sanz M.T., Higginbotham C.L., Lyons J.G. (2025). Investigation of Fenbendazole Solubility Using Particle Size Reduction Methods in the Presence of Soluplus^®^. Pharmaceutics.

[14] Zhang J., Huang C., Chen J., Xu R. (2019). Equilibrium Solubility Determination and Modeling of Fenbendazole in Cosolvent Mixtures at (283.15–328.15) K. J. Chem. Eng. Data.

[15] Bezerra G.S.N., de Lima T.A.D.M., Colbert D.M., Geever J., Geever L. (2022). Formulation and Evaluation of Fenbendazole Extended-Release Extrudes Processed by Hot-Melt Extrusion. Polymers.

[16] Zhang Y., Wischke C., Mittal S., Mitra A., Schwendeman S.P. (2016). Design of Controlled Release PLGA Microspheres for Hydrophobic Fenretinide. Mol. Pharm..

[17] Mankar S., Rach P.R. (2018). Solubility Enhancement of Poor Water Soluble Drugs by Solid Dispersion: A Review. J. Drug Deliv. Ther..

[18] Bezerra G.S.N., Colbert D.M., O’Donnell C., Cao Z., Geever J., Geever L. (2022). Compatibility Study Between Fenbendazole and Poly(Ethylene Oxide) with Application in Solid Dispersion Formulations Using Hot-Melt Extrusion. J. Pharm. Innov..

[19] Chew S.A., Arriaga M.A., Hinojosa V.A. (2016). Effects of Surface Area to Volume Ratio of PLGA Scaffolds with Different Architectures on Scaffold Degradation Characteristics and Drug Release Kinetics. J. Biomed. Mater. Res. Part A.

[20] Hussain M., Xie J., Hou Z., Shezad K., Xu J., Wang K., Gao Y., Shen L., Zhu J. (2017). Regulation of Drug Release by Tuning Surface Textures of Biodegradable Polymer Microparticles. ACS Appl. Mater. Interfaces.

[21] Pignatello R., Corsaro R. (2019). Polymeric Nanomicelles of Soluplus^®^ as a Strategy for Enhancing the Solubility, Bioavailability and Efficacy of Poorly Soluble Active Compounds. Curr. Nanomed..

[22] Shi N.-Q., Lai H.-W., Zhang Y., Feng B., Xiao X., Zhang H.-M., Li Z.-Q., Qi X.-R. (2018). On the Inherent Properties of Soluplus and Its Application in Ibuprofen Solid Dispersions Generated by Microwave-Quench Cooling Technology. Pharm. Dev. Technol..

[23] Alvarez-Rivera F., Fernández-Villanueva D., Concheiro A., Alvarez-Lorenzo C. (2016). α-Lipoic Acid in Soluplus ^®^ Polymeric Nanomicelles for Ocular Treatment of Diabetes-Associated Corneal Diseases. J. Pharm. Sci..

[24] IR Spectrum Table. https://www.sigmaaldrich.com/IE/en/technical-documents/technical-article/analytical-chemistry/photometry-and-reflectometry/ir-spectrum-table.

[25] Phillipson K., Hay J.N., Jenkins M.J. (2014). Thermal Analysis FTIR Spectroscopy of Poly(ε-Caprolactone). Thermochim. Acta.

[26] Meaurio E., Martinez de Arenaza I., Lizundia E., Sarasua J.R. (2009). Analysis of the C=O Stretching Band of the α-Crystal of Poly(L-Lactide). Macromolecules.

[27] Ramírez-Hernández A., Aguilar-Flores C., Aparicio-Saguilán A. (2019). Fingerprint Analysis of FTIR Spectra of Polymers Containing Vinyl Acetate. Dyna.

[28] Matsuura H., Miyazawa T., Machida K. (1973). Infrared Spectra of Poly(Ethylene Glycol) Dimethyl Ethers in the Crystalline State. Spectrochim. Acta Part A.

[29] Caccamo M.T., Magazù S. (2017). Ethylene Glycol—Polyethylene Glycol (EG-PEG) Mixtures: Infrared Spectra Wavelet Cross-Correlation Analysis. Appl. Spectrosc..

[30] Nejad A., Meyer E., Suhm M.A. (2020). Glycolic Acid as a Vibrational Anharmonicity Benchmark. J. Phys. Chem. Lett..

[31] Blume A., Huebner W., Messner G. (1988). Fourier Transform Infrared Spectroscopy of 13C:O Labeled Phospholipids Hydrogen Bonding to Carbonyl Groups. Biochemistry.

[32] López A., Lis M.J., Bezerra F.M., Vilaseca M., Vallés B., Prieto R., Simó M. (2019). Production and Evaluation of Antimicrobial Microcapsules with Essential Oils Using Complex Coacervation. J. Biomed. Sci. Eng..

[33] Li N., Huang C., Luan Y., Song A., Song Y., Garg S. (2016). Active Targeting Co-Delivery System Based on PH-Sensitive Methoxy-Poly(Ethylene Glycol)2K-Poly(ε-Caprolactone)4K-Poly(Glutamic Acid)1K for Enhanced Cancer Therapy. J. Colloid Interface Sci..

[34] Zhang Y., Huo M., Zhou J., Zou A., Li W., Yao C., Xie S. (2010). DDSolver: An Add-In Program for Modeling and Comparison of Drug Dissolution Profiles. AAPS J..

[35] Siepmann J., Siepmann F. (2008). Mathematical Modeling of Drug Delivery. Int. J. Pharm..

[36] Wu X.Y., Zhou Y. (1998). Finite Element Analysis of Diffusional Drug Release from Complex Matrix Systems. J. Control. Release.

[37] Schreiner T., Schaefer U.F., Loth H. (2005). Immediate Drug Release from Solid Oral Dosage Forms. J. Pharm. Sci..

[38] Upadhyay P., Chaudhary P., Upadhyay S. (2023). A Review on Formulation and Evaluation Approaches for Fast Release Tablet. Mathews J. Pharm. Sci..

[39] Quinten T., De Beer T., Almeida A., Vlassenbroeck J., Van Hoorebeke L., Remon J.P., Vervaet C. (2011). Development and Evaluation of Injection-Molded Sustained-Release Tablets Containing Ethylcellulose and Polyethylene Oxide. Drug Dev. Ind. Pharm..

[40] Jin I.S., Jo M.J., Park C.-W., Chung Y.B., Kim J.-S., Shin D.H. (2020). Physicochemical, Pharmacokinetic, and Toxicity Evaluation of Soluplus^®^ Polymeric Micelles Encapsulating Fenbendazole. Pharmaceutics.

[41] Melian M.E., Nieva C.A.B., Domínguez L., Gonzo E.E., Palma S., Bermúdez J.M. (2021). Dissolution Profiles of Fenbendazole from Binary Solid Dispersions: A Mathematical Approach. Ther. Deliv..

